# Induction of FGF23-related hypophosphatemic osteomalacia by alcohol consumption

**DOI:** 10.1016/j.bonr.2021.101144

**Published:** 2021-10-16

**Authors:** Naoko Hidaka, Hajime Kato, Minae Koga, Masaki Katsura, Yuko Oyama, Yuka Kinoshita, Seiji Fukumoto, Noriko Makita, Masaomi Nangaku, Nobuaki Ito

**Affiliations:** a Division of Nephrology and Endocrinology, The University of Tokyo Hospital, Tokyo, Japan; b Department of Radiology, The University of Tokyo Hospital, Tokyo, Japan; c Department of Internal Medicine, Koseiren Sanjo General Hospital, Niigata, Japan; d Fujii Memorial Institute of Medical Sciences, Institute of Advanced Medical Sciences, Tokushima University, Tokushima, Japan

**Keywords:** Fibroblast growth factor 23, Alcohol, Osteomalacia, Phosphate, Hypophosphatemia

## Abstract

**Context:**

Fibroblast growth factor (FGF) 23 is a hormone that regulates serum phosphate levels, the excess action of which causes chronic hypophosphatemic rickets/osteomalacia. To date, there are only two identified causes of acquired FGF23-related hypophosphatemic osteomalacia: tumor-induced osteomalacia (TIO) and osteomalacia induced by the intravenous infusion of some forms of iron preparations. In the current study, two cases of FGF23-related hypophosphatemia probably induced by chronic alcohol consumption were first introduced.

**Case description:**

Case 1 and case 2 had been drinking high amounts of alcohol for more than twenty years until they were admitted to the hospital. Case 1 was a 43-year-old man with progressive worsening multiple pains and muscle weakness who exhibited chronic hypophosphatemia with increased intact FGF23 levels. A week after admission, the serum phosphate level recovered to the reference range, and the intact FGF23 level declined. Case 1 resumed drinking after discharge, and hypophosphatemia concomitant with high intact FGF23 levels recurred. The alleviation of FGF23-related hypophosphatemia was observed each time he temporarily abstained from drinking for a short period. Case 2 was a 60-year-old man with recurrent fractures and exacerbation of pain in multiple joints who also exhibited hypophosphatemia with increased intact FGF23 levels. After admission, the serum phosphate level gradually increased to the lower limit of the normal range. The intact FGF23 level decreased, but it was still higher than 30 pg/ml, and causative FGF23-producing tumors were not identified even with thorough examinations, including somatostatin receptor scintigraphy, fluorine-18-fluorodeoxyglucose-positron emission tomography/computed tomography (^18^F-FDG-PET/CT) and systemic venous FGF23 sampling. He completely abstained from alcohol after discharge. Along with the serum phosphate level, intact FGF23 was subsequently decreased and had been normalized for 5 months. Both patients had no genetic mutation related to hereditary FGF23-related hypophosphatemic rickets/osteomalacia, including autosomal dominant hypophosphatemic rickets/osteomalacia (ADHR).

**Conclusion:**

Two cases of FGF23-related hypophosphatemia probably induced by alcohol were first introduced in this study. Identifying this reversible condition among acquired FGF23-related hypophosphatemic osteomalacia is critical to obtain better patient outcomes and save medical resources. This condition is similar to iron infusion-induced FGF23-related hypophosphatemia in terms of the dysregulation of FGF23 due to exogenous factors. Future research to elucidate the precise mechanism of these conditions is warranted.

## Highlights

• Two adults with acquired FGF23-related hypophosphatemic osteomalacia were studied.
• Tumor-induced osteomalacia was suspected initially, but no tumor was identified.
• Cessation of alcohol led to recovery from FGF23-related hypophosphatemia.
• Alcohol is a well-recognized cause of hypophosphatemia, but osteomalacia is uncommon.
• Alcohol-induced FGF23-related osteomalacia is a totally new and distinct phenomenon.

## 1 Introduction

Fibroblast growth factor (FGF) 23 is an osteoblast/osteocyte-derived phosphate-regulating hormone identified as a causative protein for autosomal dominant hypophosphatemic rickets/osteomalacia (ADHR) and tumor-induced osteomalacia (TIO) (Minisola et al., 2017; Shimada et al., 2001; White et al., 2000). Subsequent studies have revealed the physiological roles of FGF23 in regulating the serum phosphate levels. FGF23 binds to heterodimeric complexes of FGF receptor 1 (FGFR1) and alpha Klotho in the renal tubule to downregulate the expression of sodium-phosphate cotransporters (NaPi2a and NaPi2c), thereby suppressing phosphate reabsorption (Shimada et al., 2004b, Shimada et al., 2004a; Urakawa et al., 2006). FGF23 also downregulates 1 alpha-hydroxylase, upregulates 24 hydroxylase in the renal proximal tubule and decreases the active 1,25-hydroxyvitamin D: 1,25(OH)_2_D, leading to decreased intestinal absorption of phosphate (Shimada et al., 2004a). In summary, FGF23 lowers the level of serum phosphate and regulates it within the physiological range *via* the sensing mechanism of serum phosphate through FGFR1 in osteoblasts/osteocytes (Takashi et al., 2019). In addition to ADHR and TIO, currently, many diseases have been characterized by hypophosphatemia with inappropriately high secretion of FGF23, collectively known as FGF23-related hypophosphatemia, such as X-linked or autosomal recessive hypophosphatemic rickets/osteomalacia (XLH/ARHR), McCune-Albright syndrome, and linear nevus sebaceous syndrome (Avitan-Hersh et al., 2014; Feng et al., 2006; Jonsson et al., 2003; Levy-Litan et al., 2010; Lorenz-Depiereux et al., 2010, Lorenz-Depiereux et al., 2006; Riminucci et al., 2003; Yamazaki et al., 2002). The major cause of acquired FGF23-related hypophosphatemia is TIO. However, in rare cases, the intravenous administration of saccharated ferric oxide, ferric polymaltose and carboxymaltose was reported from our laboratory and others to cause FGF23-related hypophosphatemia (Schouten et al., 2009; Shimizu et al., 2009; Wolf et al., 2013). Thus, acquired cases of FGF23-related hypophosphatemic osteomalacia without iron infusion therapy were presumed to be TIO with FGF23 producing tumors mainly in the bone or soft tissue, defined as phosphaturic mesenchymal tumors (PMTs) (Folpe, 2019; Folpe et al., 2004). Frequently, clinicians could not specify the region of interest to localize FGF23-producing tumors because they could be tiny and develop anywhere in the bone or soft tissue. Therefore, systemic functional localizing techniques, including somatostatin receptor scintigraphy (^111^Indium-pentetreotide), somatostatin receptor positron emission tomography/computed tomography (PET/CT) (^68^Gallium-DOTATATE/DOTATOC/DOTANOC-PET/CT) and/or systemic FGF23 venous sampling, are usually adopted to localize PMT in cases without obvious tumors in the bone or soft tissue (Chong et al., 2013; Clifton-Bligh et al., 2013; Ito et al., 2010; Meyer et al., 2019; Nguyen and Wang, 1999; Takeuchi et al., 2004). Using somatostatin receptor PET/CT has been limited in several countries and is currently unavailable in Japan.

In this study, we report two unrelated patients with acquired FGF23-related hypophosphatemic osteomalacia who exhibited normal serum phosphate and FGF23 levels after alcohol abstinence.

## 2 Materials and methods

### 2.1 FGF23 assay and systemic venous sampling of FGF23

**Fig. 1:**
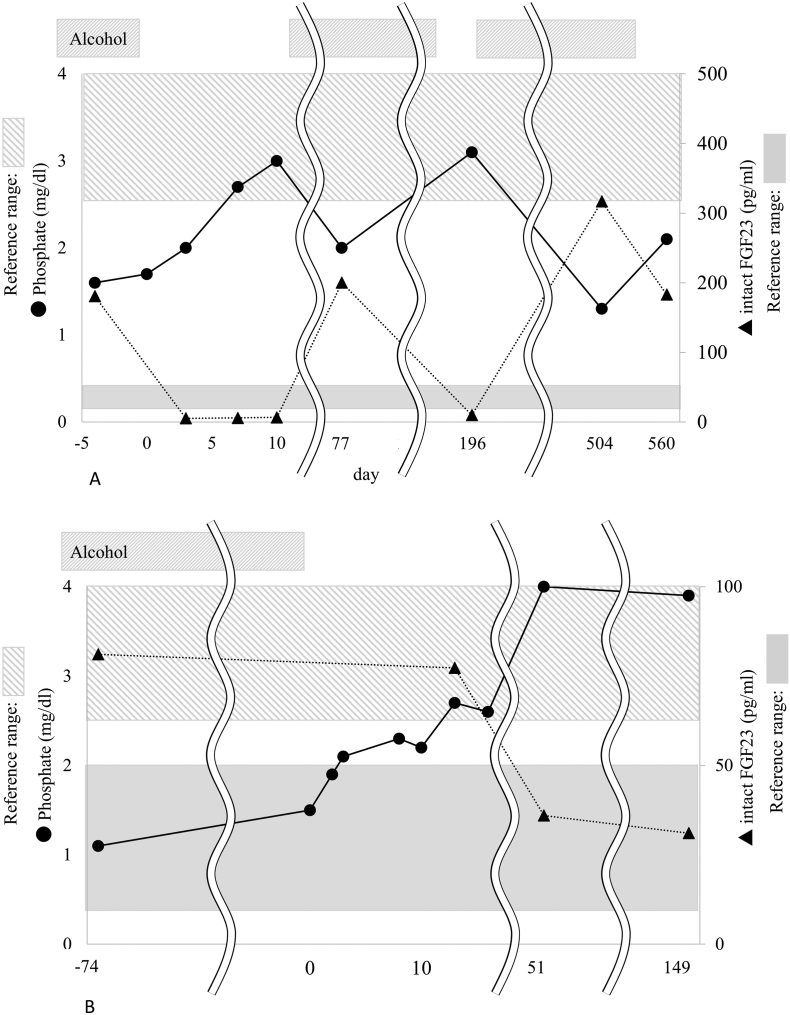
Time course of changes in the serum phosphate and FGF23 levels of the two patients. (A) represents case 1, and (B) represents case 2. The patients started to abstain from alcohol on Day 0; however, case 1 resumed drinking on Day 10. Case 1 temporarily stopped drinking from Day 186 to 196 and Day 550 to 560. In the graph areas, slashed bands represent the reference range of serum phosphate, and monotonous gray bands represent the reference range of intact FGF23.

The FGF23 assay used for both cases was performed using the FGF-23 ELISA Kit® (KAINOS Laboratories, Inc., Tokyo, Japan). Some of the samples from case 2 (on Day −74 and Day 51; Table 1 and Fig. 1B) were measured subjected to the chemiluminescent enzyme immunoassay: CLEIA kit (Deteminer CL®; Showa Denko Materials, Tokyo, Japan) using a fully automated CLEIA system (CL-JACK® NX; Showa Denko Materials, Tokyo, Japan). Both assays (ELISA and CLEIA kit) performed in this study only detected active full-length FGF23.

**Table 1:** Clinical background and laboratory data of the two patients.

	Reference ranges	Case 1			Case 2				
Age, sex		43, M			60, M				
Weight (kg)		76.2			64.0				
Height (cm)		170.0			173.0				
BMI (kg/m^2^)		26.4			21.4				
Alcohol consumption (g of pure ethanol/day, unit/day)		100, 10			100, 10				
Smoking status		Current smoker			Ex-smoker				
Onset age of osteomalacia		38			58				
		Day -4– 0	Day 10	Day 77	Day -74	Day 0	Day 13	Day 51	Day 149
Alcohol		On	Off	On	On	On	Off	Off	Off
Phosphate (mg/dl)	2.5–4.6	1.7	3.0	2.0	1.1	1.5	2.7	4.0	3.9
Corrected total calcium (mg/dl)	8.4–9.7	8.3	8.6	8.5	9.1	8.7	9.0	9.4	9.2
ALP (U/l)	38–113	217	192	291	266	189	160	182	147
BAP (μg/l)	3.7–20.9	117.0	N.E.	217.0	46.2	N.E.	N.E.	N.E.	36.1
Intact PTH (pg/ml)	15–65	98	54	43	59	90	82	39	65
1,25(OH)₂D (pg/ml)	20.0–60.0	16.0	245.0	22.0	37.0	N.E.	N.E.	40.0	40.0
25(OH)D (ng/ml)	30.0-	N.E.	N.E.	N.E.	4.7	N.E.	N.E.	6.8	7.4
TmP/GFR (mg/dl)	2.5–4.5	1.1	3.3	1.4	0.9	0.8	N.E.	3.3	3.9
Intact FGF23 (pg/ml)	10–50	181	7	200	81	N.E.	77	36	31.1
Creatinine (mg/dl)	0.65–1.07	0.68	0.73	0.66	0.76	0.67	0.67	0.82	0.82
Albumin (g/dl)	3.9–4.9	4.4	4.4	4.3	3.8	3.3	3.6	4.3	4.5
Hemoglobin (g/dl)	13.7–16.8	15.4	15.1	15.1	14.4	12.4	12.9	14.4	15.3
Hematocrit (%)	40.7–50.1	46	44.1	45	41.4	35.9	38.9	44.3	46.4
AST (U/l)	13–30	57	27	26	92	115	87	40	28
ALT (U/l)	10–42	83	34	18	33	45	51	32	21
GGT (U/l)	13–64	158	108	64	747	1273	637	167	86
Iron (μg/dl)	40–188	N.E.	N.E.	N.E.	N.E.	190	149	201	139
Ferritin (ng/ml)	14.4–303.7	N.E.	N.E.	N.E.	N.E.	2956.0	2244	684.0	392.0
Bone mineral density (T-score)									
Lumbar spine (L2-L4)		−0.1	N.E.	N.E.	N.E.	−2.2	N.E.	N.E.	−1.8
Left femoral neck		N.E.	N.E.	N.E.	N.E.	−2.0	N.E.	N.E.	−2.0

The patients started to abstain from alcohol on Day 0; however, case 1 resumed drinking on Day 10. N.E. = Not examined; ALP: alkaline phosphatase; BAP: bone-specific alkaline phosphatase; PTH: parathyroid hormone; TmP/GFR: tubular maximum transport of phosphate corrected for the glomerular filtration rate; AST: aspartate aminotransferase; ALT: alanine aminotransferase; GGT: gamma-glutamyl transpeptidase.

Systemic venous sampling of FGF23 to localize PMT was performed for case 2 (on Day 13). A catheter was inserted through the right femoral vein, and blood samples were taken from 26 sites, including the bilateral internal jugular veins, bilateral brachial veins, bilateral subclavian veins, bilateral brachiocephalic veins, superior vena cava distal, superior vena cava proximal, azygos vein, inferior vena cava proximal, inferior vena cava distal, bilateral renal veins, bilateral common iliac veins, bilateral internal iliac veins, bilateral external iliac veins, bilateral femoral vein proximal, bilateral femoral vein distal, and right hepatic vein. An experienced radiologist was engaged in the process. No complications occurred related to the sampling method. FGF23 values were measured using the ELISA kit as mentioned above.

### 2.2 Imaging

Whole-body somatostatin receptor scintigraphy was performed using a gamma camera system (E. CAM Signature; Siemens, Toshiba/Siemens, Tokyo, Japan) 4, 24, and 48 h after the injection of 199.2 MBq of ^111^In-pentetreotide, in case 2. Fluorine-18-fluorodeoxyglucose-positron emission tomography/computed tomography (^18^F-FDG-PET/CT) for the whole body was performed in accordance with the latest standard PET imaging protocols published by the Japanese Society of Nuclear Medicine 2 h after the injection of 204 MBq of ^18^F-fluorodeoxyglucose, in case 2.

### 2.3 Genetic tests

In two cases, genomic DNA was extracted from 200 μl of peripheral blood using a DNA isolation kit (NucleoSpin® Blood; Macherey-Nagel, Düren, Germany) according to the manufacturer's instructions. Whole genome sequencing was performed using the Illumina NovaSeq 6000 platform (Illumina, Inc., San Diego, CA) following standard protocols by Novogene Co., Ltd. (Beijing, China). Mutations were thoroughly identified, such as large deletions located in the exome regions and intron-exon boundaries of 18 genes related to hereditary FGF23-related disorders (*ANKH*, *DMP1*, *ENPP1*, *FAM20C*, *FGF1*, *FGF23*, *FGFR1*, *FGFR2*, *FGFR3*, *FGFR4*, *GALNT3*, *GNAS*, *HRAS*, *KRAS*, *NF1*, *NRAS*, *PHEX*, and *PTH1R*) and 43 genes related to the development of autoimmune disorders (*AIRE*, *BACH2*, *CARD9*, *CCR6*, *CD40*, *CD28*, *CTLA4*, *ERAP1*, *ERAP2*, *FCGR2A*, *FOXP3*, *ICOS*, *ICOSLG*, *IFIH1*, *IL2*, *IL21*, *IL2RA*, *IL2RB*, *IL10*, *IL12B*, *IL12A*, *IL18RAP*, *IL23R*, *IL27*, *IRF4*, *IRF5*, *IRF7*, *IRF8*, *JAK2*, *NFKB1*, *PRDM1*, *PTGER4*, *PTPN2*, *PTPN22*, *REL*, *STAT3*, *STAT4*, *TAGAP*, *TNFAIP3*, *TNIP1*, *TYK2*, *UBE2L3*, and *ZMIZ1*) were thoroughly screened.

### 2.4 Ethics approval

All procedures involving the participants in the current study were performed in accordance with the ethical standards of the institutional and/or national research committee and with the 1964 Helsinki declaration and its later amendments or comparable ethical standards. The written informed consents of the participants were obtained. The study protocols were approved by the institutional review board of the University of Tokyo (approval number: 11221, G10115, P2017016).

## 3 Case report

The clinical background and laboratory measurements at the different time points with or without alcohol consumption are shown in Table 1. The time courses of serum phosphate and FGF23 in these cases are shown in Fig. 1A (case 1) and 1B (case 2). The day of admission was defined as “Day 0” in Table 1 and Fig. 1.

### 3.1 Case 1

A 43-year-old man with pain in the hip joints and femora was referred to our division by an orthopedist at the same hospital. Starting in his twenties, he habitually drank 500 ml of spirits with 20–30% alcohol (100 g of pure ethanol, 10 units) per day. At the age of 38 years, he had pain in the left rib when he woke up. Approximately one year later, lower back pain developed when he lifted heavy items that eventually prevented him from walking. Vitamin D deficiency and Paget's disease of the bone were suspected because of high alkaline phosphatase activity and enhanced uptake in bone scintigraphy. However, the initiation of calcium and alfacalcidol did not alleviate his pain. Two months before he visited our hospital, coxalgia and femoral pain were aggravated, and he was encouraged to visit our hospital for a detailed examination. Based on suspected FGF23-related hypophosphatemic osteomalacia from his progressively worsening multiple pains with muscle weakness and the history of high alkaline phosphatase activity, a blood test was performed at the orthopedic division, revealing hypophosphatemia with inappropriately high intact FGF23 levels. Four days later, he was admitted to our department to examine the cause of FGF23-related hypophosphatemia. Repeated laboratory data revealed hypophosphatemia, hypocalcemia, and high alkaline phosphatase activity with inappropriately suppressed 1,25(OH)_2_D. Impaired renal tubular phosphate reabsorption was also detected with 1.1 mg/dl of tubular maximum transport of phosphate corrected for the glomerular filtration rate (TmP/GFR). Three days after admission, the serum phosphate level increased slightly. A week later, the serum phosphate level and TmP/GFR were completely normalized and intact FGF23 level declined in parallel (Table 1, Fig. 1A). Concurrently, the levels of hepatic enzymes (aspartate aminotransferase (AST), alanine aminotransferase (ALT), gamma-glutamyl transpeptidase (GGT)) decreased from high values (Table 1). Because hypophosphatemia was alleviated spontaneously, he was discharged on Day 10. Additionally, because the association between alcohol consumption and the development of hypophosphatemia was suspected, case 1 was advised to abstain from alcohol following discharge; however, he resumed drinking. Two months later (Day 77), laboratory data revealed the recurrence of hypophosphatemia and high intact FGF23 level. Thereafter, he temporarily abstained from alcohol for approximately 10 days after the aggravation of coxalgia and femoral pain. On each occasion, an increase in serum phosphate levels and decreased intact FGF23 levels were observed (Day 196 and Day 560; Fig. 1A).

### 3.2 Case 2

**Fig. 2:**
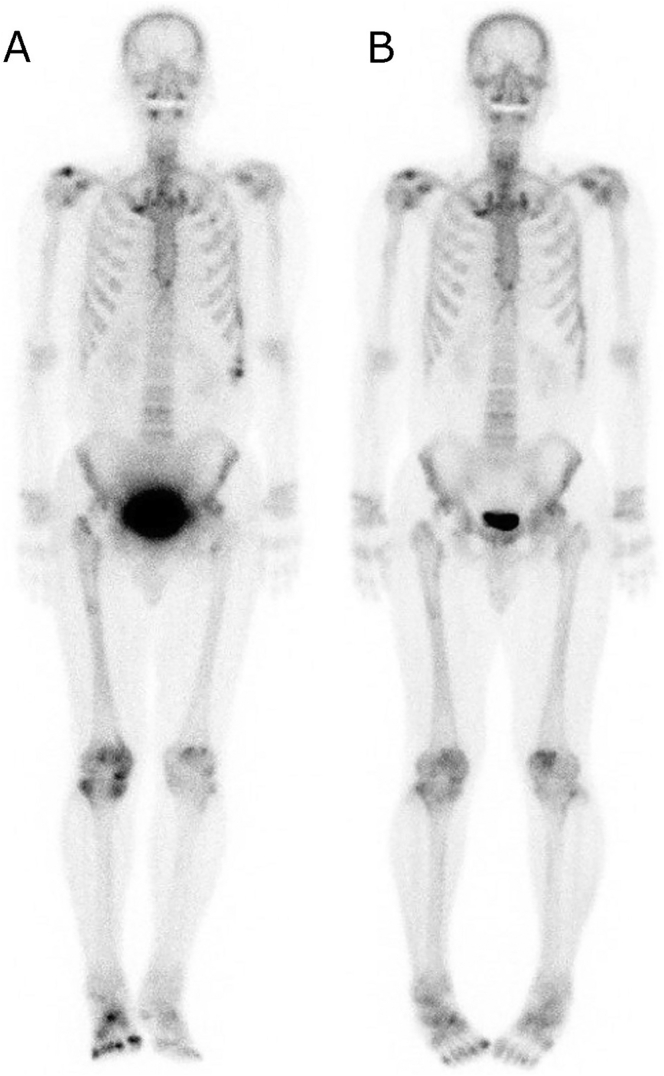
^99m^Tc bone scintigraphy in case 2. (A) was performed on Day 7. Approximately 5 months after the cessation of alcohol, (B) was performed. Uptake by multiple bone sites, including the scapula, humerus, ribs, spine, ilium, femur, tibia, metatarsal and phalanx, was revealed, indicating pseudofractures at the sites. Mild decreases in the number and amounts of uptake are shown in (B) compared with (A).

**Fig. 3:**
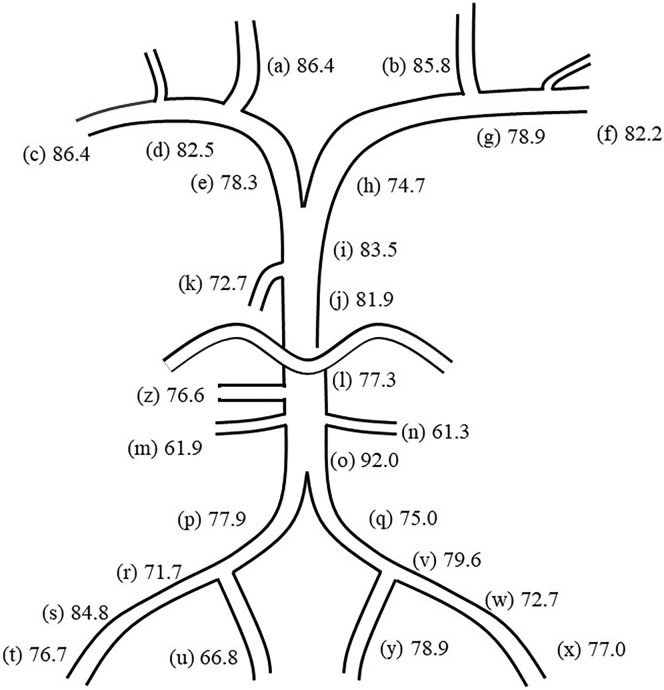
Systemic venous sampling of FGF23 in case 2. The serum samples were taken at the following twenty-six sites (a) to (z), and FGF23 measurements (pg/ml) for each site were indicated, revealing no region with outstanding FGF23 values suggesting the location of FGF23-producing tumors: (a) right (Rt.) internal jugular vein, (b) left (Lt.) internal jugular vein, (c) Rt. brachial vein, (d) Rt. subclavian vein, (e) Rt. brachiocephalic vein, (f) Lt. brachial vein, (g) Lt. subclavian vein, (h) Lt. brachiocephalic vein, (i) superior vena cava distal, (j) superior vena cava proximal, (k) azygos vein, (l) inferior vena cava proximal, (m) Rt. renal vein, (n) Lt. renal vein, (o) inferior vena cava distal, (p) Rt. common iliac vein, (q) Lt. common iliac vein, (r) Rt. external iliac vein, (s) Rt. femoral vein proximal, (t) Rt. femoral vein distal, (u) Rt. internal iliac vein, (v) Lt. external iliac vein, (w) Lt. femoral vein proximal, (x) Lt. femoral vein distal, (y) Lt. internal iliac vein, (z) Rt. hepatic vein.

Case 2 was a 60-year-old man with recurrent fractures from two years before he presented to our hospital. He had been drinking 1000 ml of beer with approximately 5% alcohol and 300 ml of spirits with 20–30% alcohol (100 g of pure ethanol, 10 units) per day since the age of twenty years. Two years before he visited our department, he felt pain in the right hip joint. An orthopedist diagnosed him with osteonecrosis of the right femoral head using an imaging test. Total hip arthroplasty was performed; however, he had fractures around the implant during the surgery. Six months before his first visit to our hospital, he had pain in his right femur and was diagnosed with a fracture of the medial condyle of the right femur. He resigned from his position at the security company because of severe pain. Immediately after that, he developed pain in the bilateral shoulders, left knee, and right foot. Osteomalacia was finally suspected as his underlying condition from the recurrent fractures and exacerbating pain in multiple joints. An additional blood test revealed hypophosphatemia with high intact FGF23 level. Therefore, he was referred to our division and admitted to examine the cause of FGF23-related hypophosphatemia. On admission, he was treated with inorganic phosphate and alfacalcidol. At that time, he had right coxalgia, bilateral knee pain, and right foot pain. The laboratory data indicated hypophosphatemia, high alkaline phosphatase activity, and elevated urinary excretion of phosphate detected as lowered TmP/GFR. Hepatic dysfunction was also indicated by high levels of hepatic enzymes (AST, ALT, GGT), high ferritin levels, mild anemia (Table 1), mild hyperbilirubinemia, and impaired glucose tolerance (data not shown). Bone scintigraphy detected multiple pseudofractures in the scapula, humerus, ribs, spine, ilium, femur, tibia, and metatarsal, corroborating his diagnosis of osteomalacia (Fig. 2A). Intact FGF23 level remained at a high level on Day 13, while the serum phosphate level increased gradually. Because the patient had suspected TIO, ^111^In-pentetreotide scintigraphy and ^18^F-FDG PET/CT were performed, failing to identify any suspected tumor. Furthermore, systemic venous sampling of FGF23 showed no outstanding FGF23 measurement among 26 sampling points (Fig. 3). The time course of the laboratory data after admission indicated an increase in serum phosphate and improved hepatic function. Because the alleviation of FGF23-related hypophosphatemia after the cessation of alcohol consumption was expected, as observed in case 1, he was discharged without inorganic phosphate and alfacalcidol on Day 17. Thereafter, he succeeded in alcohol abstinence. The intact FGF23 level was in the reference range with normal serum phosphate levels on Day 51 and continued to Day 149 with alleviated bone pain and mild improvement of the pseudofractures (Fig. 1B, 2B; Table 1).

### 3.3 Genetics

Although an obvious association was identified between alcohol consumption and the development of FGF23-related hypophosphatemia in these two cases, given the rarity of this condition among heavy drinkers in the population, the genetic propensity for the development of the condition was presumed in these two cases. Therefore, whole genome sequencing was performed to examine the genes related to hereditary FGF23-related hypophosphatemic rickets/osteomalacia and those related to the development of autoimmune diseases. However, no relevant mutations were identified among the screened genes.

## 4 Discussion

This report is the first to describe two cases of FGF23-related hypophosphatemia probably induced by alcohol consumption. These two patients had been tentatively diagnosed with TIO because of adult-onset FGF23-related hypophosphatemic osteomalacia. Contrary to the initial prediction, their hypophosphatemia with high intact FGF23 levels was alleviated a few weeks after their admission to the hospital. Because the serum phosphate level in case 2 elevated gradually and remained lower, we performed ^111^In-pentetreotide scintigraphy, ^18^F-FDG PET/CT and systemic venous sampling of FGF23, which did not identify any causative tumor. Additionally, whole genome sequencing for these cases revealed no mutations in the recognized genes related to hereditary FGF23-related hypophosphatemic rickets/osteomalacia or autoimmune diseases. We hypothesized that a certain change derived from the hospitalized environment, such as alcohol abstinence, led to the improved FGF23-related hypophosphatemia. This finding is supported by FGF23-related hypophosphatemia in case 1 recurring after the recommencement of drinking and improved each time drinking was discontinued for a short duration; additionally, case 2, who was successful in continuous abstinence, showed no recurrence of hypophosphatemia with high intact FGF23 levels afterward.

Although the development of osteomalacia is uncommon in chronic alcoholism, mild hypophosphatemia with chronic alcoholism has been repeatedly described in the literature (Baj et al., 2020; Felsenfeld and Levine, 2012; Palmer and Clegg, 2017). Decreased expression of sodium-phosphate cotransporters NaPi2a and NaPi2c expressed in the proximal tubules was reported as a mechanism for the development of hypophosphatemia among individuals with chronic alcoholism (Baj et al., 2020; Palmer and Clegg, 2017). The expression levels of these cotransporters were reported to be downregulated by acidic conditions that chronic alcoholics occasionally develop (Curthoys and Moe, 2014). In addition to the deranged regulation in the proximal tubule, frequently accompanying conditions such as decreased dietary intake and subsequent vitamin D deficiency, the use of antacids, as well as vomiting and chronic diarrhea contribute to the development of chronic hypophosphatemia among alcoholics (Palmer and Clegg, 2017). Bone fragility in chronic alcoholics has also been reported (Kaukonen et al., 2006; Maurel et al., 2012; Schuckit, 2009). Individuals with alcoholism tend to show low bone mineral density (BMD) concomitantly with uncoupled excessive bone resorption, increased risk of fracture and chronic hypophosphatemia. Vitamin D deficiency may play roles in the development of bone disorders among alcoholics. However, to date, this issue has not been investigated or discussed in association with the action of FGF23. Only one report, to our knowledge, has revealed higher FGF23 levels among alcoholics than among healthy subjects (Quintero-Platt et al., 2017). However, in that report, FGF23 was measured using a C-terminal FGF23 assay that detects inactive C-terminal fragments of FGF23 in addition to active intact FGF23. Additionally, the association between the development of hypophosphatemia and serum FGF23 levels was not discussed in that report (Quintero-Platt et al., 2017). The present cases of FGF23-related hypophosphatemia, probably induced by alcohol, may provide new insight into the development of continued hypophosphatemia and bone metabolic disturbances among chronic heavy alcohol drinkers. It is believed that the underlying etiology for the development of hypophosphatemic osteomalacia in the currently reported cases was completely different from that of chronic hypophosphatemia frequently observed in chronic alcoholism, given its severity and obvious biochemical derangements imitating those of tumor-induced osteomalacia. To this point, the underlying mechanism by which alcohol consumption disrupts homeostasis of FGF23 and serum phosphate probably among particular patients remains to be elucidated. Furthermore, the difference in the period taken for the normalization of FGF23-related hypophosphatemia after alcohol abstinence between cases 1 and 2 suggests that the underlying mechanism of the development of FGF23-related hypophosphatemic osteomalacia may vary between individuals with chronic alcoholism.

FGF23-related hypophosphatemia in the present cases was strongly suggested to be induced by chronic heavy drinking according to the time course of the serum phosphate and FGF23 levels, which changed in parallel with alcohol consumption. Our cases were, in some ways, similar to iron infusion-induced FGF23-related hypophosphatemia, which was reported by our laboratory and others approximately a decade ago, indicating that an exogenous factor led to FGF23-related hypophosphatemia (Schouten et al., 2009; Shimizu et al., 2009; Wolf et al., 2013). Subsequent randomized controlled trials have revealed that the diverse reactions of serum FGF23 and phosphate are highly dependent on the forms of intravenous iron preparation utilized; the administration of ferric carboxymaltose led to FGF23-related hypophosphatemia in approximately 70% of the participants; whereas fermoxytol and ferric derisomaltose led its development in less than 10% of the participants (Emrich et al., 2020; Wolf et al., 2020, Wolf et al., 2018). Although the underlying mechanism for this iron infusion-induced FGF23-related hypophosphatemia remains unclear, it has already been clarified that the final rate-limiting step to determine the serum intact FGF23 level is the posttranslational O-glycosylation initiated by *N*-acetylgalactosaminyltransferase 3 (GalNAc-T3) (Frishberg et al., 2007; Takashi et al., 2019). After the translational step of FGF23 mRNA, 178 Thr in FGF23 protein is modified by mucin-type O-glycosylation, which is initiated by GalNAc-T3, to protect FGF23 protein from cleavage. With this mechanism, the level of serum intact FGF23 is modulated by the serum phosphate level (Frishberg et al., 2007; Takashi et al., 2019). For that reason, disruption of O-glycosylation must be involved in the development of intravenous iron preparation-induced FGF23-related osteomalacia. In contrast, increased transcription of FGF23 due to iron deficiency was previously shown to be attributed to the activation of hypoxia-inducible factor 1 alpha (Edmonston and Wolf, 2020). Therefore, first, pre-existing iron deficiency upregulates the transcription of FGF23 through activated hypoxia-inducible factor 1 alpha and/or erythropoietin production (Edmonston and Wolf, 2020). Second, some specific iron preparations, such as ferric carboxymaltose, are proposed to enhance the initiation of O-glycosylation irrelevant to the actual serum phosphate level, resulting in inappropriately high intact FGF23 levels with concomitant hypophosphatemia (Wolf et al., 2013). We explored several possible underlying genetic conditions in our patients which increase the transcription of *FGF23* and explain this ‘two-hit’ hypothesis. First, we ruled out the presence of a milder form of ADHRs. A previous report indicated that both C-terminal and intact FGF23 were elevated in ADHR patients with iron deficiency, while normal subjects with iron deficiency showed an increase only in C-terminal FGF23 but not in intact FGF23 (Imel et al., 2011). Because FGF23 protein in ADHR patients is resistant to cleavage, the intact FGF23 levels were high under conditions of increased transcription of *FGF23* stimulated by iron deficiency (Imel et al., 2011). Alcoholics, particularly those with liver dysfunction, show higher C-terminal FGF23 levels (Quintero-Platt et al., 2017). Therefore, an increase in the intact FGF23 levels can develop in response to alcohol consumption if the two participants in this study had ADHRs or other relevant genetic conditions where the degradation of FGF23 was impaired. However, the whole genome sequencing data contrasted our expectations: no mutations in *FGF23* responsible for ADHR, and in the other genes related to the development of other hereditary FGF23-related hypophosphatemic rickets/osteomalacia. Second, we explored the possible involvement of autoimmunity. The genes related to the development of autoimmune disorders were also analyzed because of the case report of autoimmune tumoral calcinosis due to spontaneously developed anti-FGF23 neutralizing antibody (Roberts et al., 2018). Although tumoral calcinosis was caused by the impaired function of intact FGF23, mirroring the image of FGF23-related osteomalacia, we hypothesized that a case is also possible in which an autoantibody is developed that stimulates the secretion of intact FGF23. However, no apparent function-affecting mutations were identified among the analyzed relevant genes. In addition to the listed relevant genes, pathogenic or likely pathogenic homozygous mutations or recognized heterozygous dominant negative mutations were thoroughly screened, and no such mutation was detected.

Elucidating the main source of intact FGF23 secretion in these cases of FGF23-related hypophosphatemic osteomalacia probably induced by alcohol is also intriguing. There has been only one report that revealed that alcoholics, particularly those with liver cirrhosis, showed elevated C-terminal FGF23, although intact FGF23 was not measured in that study. Therefore, liver dysfunction might increase the transcription of FGF23 through an unknown mechanism (Quintero-Platt et al., 2017). Furthermore, ectopic FGF23 production was identified in a patient with FGF23-related hypophosphatemic rickets with biliary atresia (Wasserman et al., 2016). Some studies have indicated the ectopic upregulated transcription of *FGF23* in the heart and kidney under pathological conditions (Andrukhova et al., 2015; Smith et al., 2017; Spichtig et al., 2014; Van Venrooij et al., 2014; Zanchi et al., 2013). However, in the current study, the liver was unlikely to be the source of FGF23 in case 2 because the FGF23 level at the hepatic vein in systemic FGF23 sampling was comparable to that at the other sampling points. According to the unanimously upregulated intact FGF23 at all sampling points in the systemic FGF23 sampling, intact FGF23 was supposed to be inappropriately secreted from osteoblasts/osteocytes in case 2.

This study has some limitations. In particular, in case 2, because he strictly abstained from alcohol, the effect of alcohol consumption on the development of FGF23-related hypophosphatemia was not reconfirmed to date.

In conclusion, two cases of FGF23-related hypophosphatemic osteomalacia probably induced by alcohol consumption were first introduced in this study. We believe this reversible condition is worth noting to avoid invasive and costly investigations, including scintigraphy, PET/CT and systemic venous sampling, when alcohol-induced FGF23-related hypophosphatemic osteomalacia is confirmed after alcohol abstinence. The results in the current study indicated a new entity of acquired FGF23-related hypophosphatemia other than TIO and iron infusion-induced FGF23-related hypophosphatemia. In the future, further investigation is warranted concerning the precise mechanism of the development of FGF23-related hypophosphatemia in limited patients with massive alcohol consumption.

## Funding

This study received no specific grants from funding agencies in the public, commercial, or not-for-profit sectors.

## Declaration of competing interest

All authors state they have nothing to disclose.
